# Association of karyomegalic interstitial nephritis with focal segmental glomerulosclerosis

**DOI:** 10.4322/acr.2021.343

**Published:** 2021-11-12

**Authors:** Momal Tara Chand, Awais Zaka, Hong Qu

**Affiliations:** 1 Ascension St John Hospital, Department of Pathology, Detroit, Michigan, USA; 2 Ascension St John Hospital, Department of Nephrology, Detroit, Michigan, USA

**Keywords:** Glomerulosclerosis, Focal Segmental, Kidney, Nephritis, Interstitial

## Abstract

Karyomegalic interstitial nephritis (KIN), first described in 1974, is a rare form of chronic tubulointerstitial nephritis. It is defined by the presence of markedly enlarged, hyperchromatic nuclei with prominent nucleoli, mainly involving tubular epithelial cells of the kidney, accompanied by marked interstitial fibrosis. The disease presents as asymptomatic proteinuria, gradually progresses to chronic kidney disease and eventually leads to end-stage renal disease by 30-40 years. The etiology of the disease remains unclear; however, genetic risk factors and possible association with HLA (B27/35) is proposed by some. It has also been linked to *FAN1* (FANCD2/FANC1- associated nuclease 1) mutation.

**Case Report:**

We present two cases of KIN with associated focal segmental glomerulosclerosis. Both patients presented with nephrotic range proteinuria. The biopsies demonstrated marked enlargement of tubular nuclei (3-5x larger than the uninvolved tubular nuclei, a metric used by some authors in previous studies) in some tubules, meeting the diagnostic criteria of KIN.. Interestingly, case one had a prior biopsy that showed minimal change disease. In the biopsies done at our institution, H&E sections showed patchy tubular attenuation with readily recognizable tubular cell mitotic figures, indicating concurrent acute tubular injury. Electron microscopy showed diffuse podocyte foot process effacement, along with microvillous transformation, podocyte hypertrophy, and cytoplasmic vacuoles, suggesting podocyte injury. This cytoplasmic vacuolization was also observed in the tubular epithelial cells. In both cases, the injury factor appeared to target both podocytes and tubular cells.

## INTRODUCTION

Karyomegalic interstitial nephritis (KIN), a rare and uncommon hereditary cause of chronic interstitial nephritis, was first described over 40 years ago.^1^ This disease has a prevalence of less than 1% and has no defined treatment guidelines.^2^ It is characterized by progressive renal failure, proteinuria, and a history of recurrent respiratory infections.^1^ Histologically, KIN reveals hyperchromatic and enlarged nuclei of tubular epithelial cells accompanied by marked interstitial fibrosis.^1^ The pathogenesis of KIN remains unclear, although some believe that viral infections and/or environmental toxins play a role. Genetic risk factors and possible association with HLA (B27/35) have also been proposed by some. It has also been linked to *FAN1* (FANCD2/FANC1- associated nuclease 1) mutation.

Herein, we report two cases KIN with associated glomerulosclerosis. Both patients presented with nephrotic range proteinuria. The biopsies demonstrated typical features of KIN with marked enlargement of tubular nuclei in some tubules (3-5x larger than the uninvolved tubular nuclei).

## CASE REPORTS

### Case 1:

A 28-year-old Asian male was sent by his primary care physician for abnormal labs, potassium of 2.8 mmol/L (reference range 3.6 to 5.2 millimoles per liter (mmol/L), and creatinine of 4.03 mg/dl (reference range 0.7-1.2 milligram per deciliter). His past medical history was significant for minimal change disease diagnosed three years prior. He had taken prednisone and then tacrolimus without significant improvement in proteinuria. At the time of the presentation, he was not using steroids or tacrolimus. He was only using bumetanide and metolazone. He denied any family history of kidney disease. A physical exam showed bilateral 2+ pitting edema of the lower extremity. The patient was afebrile, and other vitals were stable. Laboratory values revealed a white blood cell (WBC) of 11.8K/mcL (reference range: 5 to 10 k/mcl), hemoglobin of 11 gm/dL (reference range 13.5 to 15.5 grams per deciliter), platelets of 518K/mcL (reference range 150,000 to 450,000 per microliter of blood), blood urea nitrogen (BUN) of 21 mg/dL (reference range 7 to 20 milligram per deciliter), and creatinine of 3.97 mg/dL (reference range 0.7-1.2 milligram per deciliter). The patient’s baseline creatinine two years prior to this encounter was 1.0 mg/dL (reference range 0.7-1.2 milligram per deciliter). His urinalysis was positive for proteinuria and glucosuria. Liver function tests were normal. His urine protein to creatinine ratio (UPCR) was 16.7.

**Figure 1:**
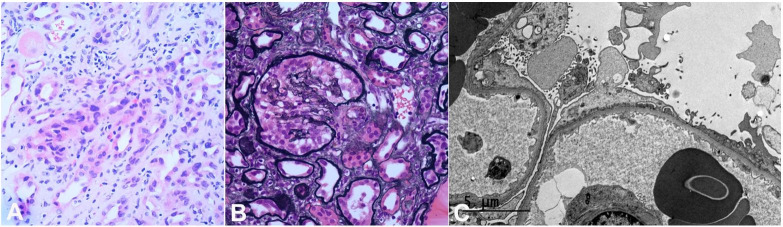
**A** – Light microscopy showing enlarged and hyperchromatic nuclei in some tubules (H and E ×200); **B** – The glomeruli exhibiting podocyte hyperplasia (Jones methenamine silver satin, 200X); **C** – Electron microscopy showing diffuse foot process effacement with microvillous transformation.

All labs for acute kidney injury and nephrotic syndrome, including antinuclear antibody (ANA), anti-double-stranded DNA, Hepatitis B, and C serology, C3 and C4 complement levels, and creatine phosphokinase (CPK) level were negative or within normal limits. An ultrasound showed increased echogenicity throughout the substance of right and left kidneys with increased cortical medullary differentiation, all compatible with medical renal disease. The right kidney measured 10.6 cm and the left measured 10.1 cm. No mass or hydronephrosis was identified in either of the kidneys. Proteinuria and glucosuria led to the suspicion of Fanconi’s syndrome, possibly from tubular injury due to high protein load. A kidney biopsy showed 33 glomeruli under light microscopy, three of which were globally sclerotic and three showed podocyte proliferation. It also showed significant interstitial fibrosis, tubular atrophy, and multiple foci of tubules with enlarged and hyperchromatic nuclei with associated patchy to diffuse interstitial infiltrates of mononuclear inflammatory cells (Figure 1A and B),

Immunofluorescence was negative for albumin, fibrinogen, C1q, C3, C4, IgA, IgG, IgM, kappa, and lambda light chain stains. Electron microscopy was notable for widespread podocyte foot process fusion (95-100%) and focal microvillous transformation (Figure 1C).

Many podocytes appeared enlarged with cytoplasmic vacuoles. The mesangial areas were unremarkable. The glomerular basement membrane (GBM) was relatively uniform and of normal thickness. No electron-dense deposits or organized protein deposits were present.

The patient was started on prednisone 60mg per oral (PO) daily and was told to follow up with nephrology. However, he was lost to follow up. He subsequently returned to the hospital with a chief complaint of chest pain. His creatinine was 20.27mg/dL and required hemodialysis.

### Case 2:

A 28-year-old Asian female came to the emergency department complaining of bilateral leg swelling and abdominal pain for three weeks. She was diagnosed with hypertension at age 22 but had no family history of hypertension or kidney disease. The patient was afebrile, and her other vitals were stable. A physical exam showed 2+ pitting edema of the bilateral lower extremity. Her laboratory values were as follows: WBC of 16.7K/mcL (reference range: 5 to 10 k/mcl), hemoglobin of 10.7gm/dL (reference range 13.5 to 15.5 grams per deciliter), platelets were 95K/mcL (reference range 150,000 to 450,000 per microliter of blood), blood urea nitrogen of 112mg/dL (reference range 7 to 20 milligram per deciliter), and creatinine of 7.32mg/dL (reference range 0.7-1.2 milligram per deciliter). Her liver enzymes were mildly elevated with an aspartate transaminase (AST) of 92U/L (reference range 5 to 40 units per liter of serum) and alanine aminotransferase (ALT) of 63U/L (reference range 7 to 55 units per liter). Her urinalysis was positive for proteinuria, glucosuria, and 12 red cells. The UPCR was 5.5.

**Figure 2:**
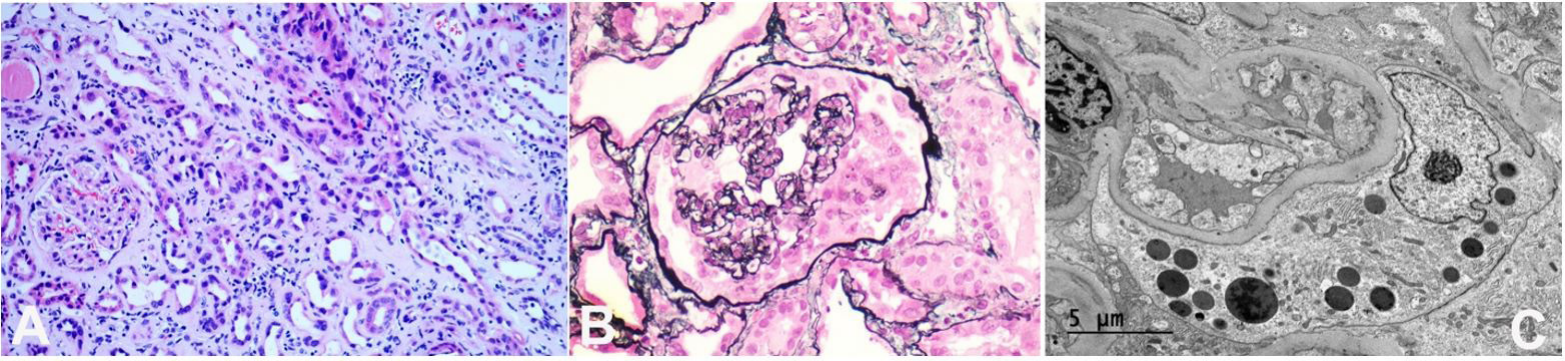
**A** – Light microscopy showing enlarged and hyperchromatic nuclei in some proximal tubules (H&E,100X); **B** – Jones methenamine silver satin showing collapse of capillary lumen and hyperplasia of podocytes (200x); **C** – Electron microscopy showing effacement of foot processes in the cytoplasm of podocytes and resorption granules.

Further workup for acute kidney injury, including ANA, anti-double-stranded DNA, antineutrophil cytoplasmic antibodies (ANCA), Hepatitis B, and C serology, C3 and C4 complement levels, and CPK level was negative or within normal limits. Her direct and indirect Coomb’s test were positive, and she was started on 100mg of prednisone by hematology for possible Evan’s Syndrome. The ultrasound showed mildly enlarged echogenic kidneys right kidney (15.2 x 5.9 x 7.4 cm) and normal left kidney (12.3 x 6.0 x 5.9 cm). There was also mild prominence of the renal collecting system on the right side. A kidney biopsy was completed. Light microscopy showed thirty glomeruli, more than ten of which showed global or segmental collapse of capillaries with the proliferation of podocytes, suggesting collapsing variant focal segmental glomerulosclerosis. No crescents were identified. Diffuse tubular alteration and edema were also noted, revealing multiple foci of tubules with enlarged and hyperchromatic nuclei, along with patchy to diffuse interstitial inflammation (Figure 2A and B). Immunofluorescence showed granular or smudgy deposits of low to moderate intensity of C1q, C3, and IgM in the capillary wall. All glomeruli were negative for albumin, fibrinogen, C4, IgA, IgG, kappa, and lambda light chain stains. Electron microscopy showed extensive widespread podocyte foot process fusion (100%) (Figure 2C). Many proliferating podocytes were enlarged with cytoplasmic vacuoles and resorption granules, filling up the urinary space. The mesangial areas were unremarkable. The glomerular basement membrane (GBM) was mildly thickened. No electron-dense deposits or organized protein deposits were present.

The patient was started on Furosemide 80 mg PO two times a day and prednisone 60mg once daily. On the day of discharge, her serum creatinine was 6.9 mg/dl, which came down to 1.5mg/dL within a month. Afterward, the patient remained non-compliant with her medicines and follow-ups. Her creatinine remained stable at Cr 1.8 - 2.0 mg/dL over the next twelve months, after which she was lost to follow up.

## DISCUSSION

KIN is a form of chronic interstitial nephritis, complete knowledge of which remains elusive. In the last two decades, however, many have attempted to explain this phenomenon. Pathologists have long before noticed karyomegalic cellular abnormalities in the liver and kidneys of animals who have been exposed to pyrrolizidine alkaloids with aflatoxin and busulphan.^3^ Burry et al.^4^ reported the case of a 22-year-old female whose autopsy revealed megalocytosis and extreme dysplasia in the liver, kidney, and pancreas, without any history of exposure to toxins. Mihatsch et al.^5^ conducted the first study, which described these megalocytic changes and associated interstitial inflammation, a unique entity—karyomegalic interstitial nephritis. In a case series, they discussed the clinical course of three patients, two brothers and a third unrelated male, who presented with progressive renal failure, and whose renal biopsy showed marked karyomegaly and chronic interstitial nephritis. This work was taken up by Spoendlin et al.,^6^ who found, in their patients as well as the patients reported by Mihatsch et al.^5^ familial clustering of human leukocyte antigen (HLA) haplotypes A9 and B35. This clustering led the researchers to suggest the possibility of a genetic defect on chromosome six, near the HLA locus. More cases of karyomegalic interstitial nephritis started to appear with closely resembling familial clustering.^7^ Then, a few cases were reported where no familial clustering was identified at all.^8^^,^^9^^,^^10^ Finally, in one case series of three patients with KIN, the author came across a completely different haplotype. However, all three patients in this paper were found to have high serum levels of Ochratoxin.

More recently, the pathogenic process has been linked to an underlying gene mutation. Zhou et al.^11^ suggested the cause of KIN to be a mutation in *FAN1* gene, a gene that encodes Fanconi anemia-associated nuclease-1 protein and helps repair DNA interstrand cross-link damage.^12^ Mutations in two other genes have also been identified in patients with renal ciliopathies, further strengthening the concept of a potential link between defective DNA repair and the pathogenesis of KIN.^13^

So far, the most plausible explanation for the pathogenesis of this disorder involves an environmental insult—viral or toxic—to a genetically susceptible individual. This process results in abnormal deoxyribonucleic acid ploidy, leading to morphological alterations in neuronal, muscular, and epithelial cells. Patients can present with renal or non-renal manifestations. The natural history of the renal disease involves presentation with slowly progressive kidney disease in the third or fourth decade of life, leading, eventually, to end-stage renal disease in early adulthood.^9^ Urine chemistry ranges from mild proteinuria, usually less than 1gm/day, to glucosuria, present in up to 75% of patients. Active urinary sediment is extremely rare and, when it does occur, consists of red cells and karyomegalic cells.^14^

Characteristic renal biopsy findings are seen on light microscopy and include non-specific but severe chronic interstitial fibrosis, along with tubular cell changes. No immunofluorescent and electron microscopic pattern peculiar to KIN has been identified. On light microscopy, tubular cells show enlarged, irregularly shaped, hyperchromatic nuclei (up to five times the size of a normal tubular epithelial cell nucleus). Atypical cells do not seem to follow a pattern or make clusters. They are seen throughout the tubules, distal or proximal, atrophic or non-atrophic.^14^ The affected cells can sometimes be so enlarged that they reduce the number of cells per tubular cross-section to one or two, mimicking an endothelial-like layer on the tubular basement membrane. Areas of chronic interstitial inflammation and fibrosis are almost always evident, which can or cannot be adjacent to affected tubules. Glomerular lesions are rare and manifest as non-specific glomerulosclerosis.

Bhandari et al.^9^ described glomerular changes which included mesangial thickening and nuclear enlargement in epithelial cells lining the Bowman’s capsule in three of six patients. In 2014, Rhada et al.^2^ presented the case of an eight-year-old male, likely the youngest patient with KIN in the literature, whose biopsy showed, typical nuclear changes of KIN in the tubular epithelial cells and segmental sclerosis in four out of 23 glomeruli. To our knowledge, this is the first case in the literature in which KIN and FSGS are described together, raising the question of a common link to the underlying genetic abnormality between KIN and FSGS. Our patients’ biopsy, too, showed tubule-interstitial changes as well as focal segmental glomerulosclerosis. The *FAN1* gene, most strongly associated with KIN, has no link with FSGS. The *FAN1* gene is located on chromosome 15, while most of the genes associated with FSGS are located on chromosome 19. Radha et al.^2^ suggested that KIN was an incidental finding, having nothing to do with FSGS.

In the last two decades, our understanding of KIN has evolved. Moving on from observations in animals and autopsies, we are now able to study the disease in the living patients, tracing its mysteries down to the genetic level, where, according to many authorities, the final code lies. We suggest some link between KIN and FSGS. Because tubular epithelial cells and podocytes originate from the same tissue, an injury at the genetic level that leads to both pathologies cannot be ruled out. Our cases call for more genetic testing in patients who present with nephrotic or nephritic syndromes at an early age.
